# Supplementary material for: Resolving the full spectrum of human genome variation using Linked-Reads
Source: Genome Res. 2019 Apr;29(4):635–45. doi: 10.1101/gr.234443.118 (PMC6442396; doi:10.1101/gr.234443.118)
Supplement: Supplemental Material [file supp_gr.234443.118_Supplemental_longranger-2.2.zip › longranger-2.2.tar.bz2!/lr2.2-public/lib/stan/cmdstan/stan/doc/api/html/ast__def_8cpp_source.html]

Stan: src/stan/gm/ast\_def.cpp Source File


|  |  |
| --- | --- |
|  | Stan  2.5.0  probability, sampling & optimization |


- Main Page
- Related Pages
- Namespaces
- Classes
- Files

- File List
- File Members

All Classes Namespaces Files Functions Variables Typedefs Enumerations Enumerator Friends Macros Pages

- src
- stan
- gm

ast\_def.cpp

Go to the documentation of this file.

1 #ifndef \_\_STAN\_\_GM\_\_AST\_DEF\_HPP\_\_

2 #define \_\_STAN\_\_GM\_\_AST\_DEF\_HPP\_\_

3

4 #include <boost/variant/apply\_visitor.hpp>

5 #include <boost/variant/recursive\_variant.hpp>

6

7 #include <cstddef>

8 #include <limits>

9 #include <climits>

10 #include <iostream>

11 #include <map>

12 #include <stdexcept>

13 #include <string>

14 #include <utility>

15 #include <vector>

16

17 #include <stan/gm/ast.hpp>

18

19 namespace stan {

20

21  namespace gm {

22

23  std::ostream& write\_base\_expr\_type(std::ostream& o, base\_expr\_type type) {

24  switch (type) {

25  case INT\_T :

26  o << "int";

27  break;

28  case DOUBLE\_T :

29  o << "real";

30  break;

31  case VECTOR\_T :

32  o << "vector";

33  break;

34  case ROW\_VECTOR\_T :

35  o << "row vector";

36  break;

37  case MATRIX\_T :

38  o << "matrix";

39  break;

40  case ILL\_FORMED\_T :

41  o << "ill formed";

42  break;

43  case VOID\_T :

44  o << "void";

45  break;

46  default:

47  o << "UNKNOWN";

48  }

49  return o;

50  }

51

52  // expr\_type ctors and methods

53  expr\_type::expr\_type()

54  : base\_type\_(ILL\_FORMED\_T),

55  num\_dims\_(0) {

56  }

57  expr\_type::expr\_type(const base\_expr\_type base\_type)

58  : base\_type\_(base\_type),

59  num\_dims\_(0) {

60  }

61  expr\_type::expr\_type(const base\_expr\_type base\_type,

62  size\_t num\_dims)

63  : base\_type\_(base\_type),

64  num\_dims\_(num\_dims) {

65  }

66  bool expr\_type::operator==(const expr\_type& et) const {

67  return base\_type\_ == et.base\_type\_

68  && num\_dims\_ == et.num\_dims\_;

69  }

70  bool expr\_type::operator!=(const expr\_type& et) const {

71  return !(\*this == et);

72  }

73  bool expr\_type::operator<(const expr\_type& et) const {

74  return (base\_type\_ < et.base\_type\_)

75  || ( base\_type\_ == et.base\_type\_

76  && num\_dims\_ < et.num\_dims\_ );

77  }

78  bool expr\_type::operator<=(const expr\_type& et) const {

79  return (base\_type\_ < et.base\_type\_)

80  || ( base\_type\_ == et.base\_type\_

81  && num\_dims\_ <= et.num\_dims\_ );

82  }

83  bool expr\_type::operator>(const expr\_type& et) const {

84  return (base\_type\_ > et.base\_type\_)

85  || ( base\_type\_ == et.base\_type\_

86  && num\_dims\_ > et.num\_dims\_ );

87  }

88  bool expr\_type::operator>=(const expr\_type& et) const {

89  return (base\_type\_ > et.base\_type\_)

90  || ( base\_type\_ == et.base\_type\_

91  && num\_dims\_ >= et.num\_dims\_ );

92  }

93  bool expr\_type::is\_primitive() const {

94  return is\_primitive\_int()

95  || is\_primitive\_double();

96  }

97  bool expr\_type::is\_primitive\_int() const {

98  return base\_type\_ == INT\_T

99  && num\_dims\_ == 0U;

100  }

101  bool expr\_type::is\_primitive\_double() const {

102  return base\_type\_ == DOUBLE\_T

103  && num\_dims\_ == 0U;

104  }

105  bool expr\_type::is\_ill\_formed() const {

106  return base\_type\_ == ILL\_FORMED\_T;

107  }

108  bool expr\_type::is\_void() const {

109  return base\_type\_ == VOID\_T;

110  }

111  base\_expr\_type expr\_type::type() const {

112  return base\_type\_;

113  }

114  size\_t expr\_type::num\_dims() const {

115  return num\_dims\_;

116  }

117

118  std::ostream& operator<<(std::ostream& o, const expr\_type& et) {

119  write\_base\_expr\_type(o,et.type());

120  if (et.num\_dims() > 0)

121  o << '[' << et.num\_dims() << ']';

122  return o;

123  }

124

125  expr\_type promote\_primitive(const expr\_type& et) {

126  if (!et.is\_primitive())

127  return expr\_type();

128  return et;

129  }

130

131  expr\_type promote\_primitive(const expr\_type& et1,

132  const expr\_type& et2) {

133  if (!et1.is\_primitive() || !et2.is\_primitive())

134  return expr\_type();

135  return et1.type() == DOUBLE\_T ? et1 : et2;

136  }

137

138  void function\_signatures::reset\_sigs() {

139  if (sigs\_ == 0) return;

140  delete sigs\_;

141  sigs\_ = 0;

142  }

143  function\_signatures& function\_signatures::instance() {

144  // FIXME: for threaded models, requires double-check lock

145  if (!sigs\_)

146  sigs\_ = new function\_signatures;

147  return \*sigs\_;

148  }

149  void

150  function\_signatures::set\_user\_defined(const

151  std::pair<std::string,

152  function\_signature\_t>&

153  name\_sig) {

154  user\_defined\_set\_.insert(name\_sig);

155  }

156  bool

157  function\_signatures::is\_user\_defined(const std::pair<std::string,

158  function\_signature\_t>&

159  name\_sig) {

160  return user\_defined\_set\_.find(name\_sig) != user\_defined\_set\_.end();

161  }

162  bool

163  function\_signatures::is\_defined(const std::string& name,

164  const function\_signature\_t& sig) {

165  const std::vector<function\_signature\_t> sigs = sigs\_map\_[name];

166  for (size\_t i = 0; i < sigs.size(); ++i)

167  if (sig.second == sigs[i].second)

168  return true;

169  return false;

170  }

171  void function\_signatures::add(const std::string& name,

172  const expr\_type& result\_type,

173  const std::vector<expr\_type>& arg\_types) {

174  sigs\_map\_[name].push\_back(function\_signature\_t(result\_type,arg\_types));

175

176  }

177  void function\_signatures::add(const std::string& name,

178  const expr\_type& result\_type) {

179  std::vector<expr\_type> arg\_types;

180  add(name,result\_type,arg\_types);

181  }

182  void function\_signatures::add(const std::string& name,

183  const expr\_type& result\_type,

184  const expr\_type& arg\_type) {

185  std::vector<expr\_type> arg\_types;

186  arg\_types.push\_back(arg\_type);

187  add(name,result\_type,arg\_types);

188  }

189  void function\_signatures::add(const std::string& name,

190  const expr\_type& result\_type,

191  const expr\_type& arg\_type1,

192  const expr\_type& arg\_type2) {

193  std::vector<expr\_type> arg\_types;

194  arg\_types.push\_back(arg\_type1);

195  arg\_types.push\_back(arg\_type2);

196  add(name,result\_type,arg\_types);

197  }

198  void function\_signatures::add(const std::string& name,

199  const expr\_type& result\_type,

200  const expr\_type& arg\_type1,

201  const expr\_type& arg\_type2,

202  const expr\_type& arg\_type3) {

203  std::vector<expr\_type> arg\_types;

204  arg\_types.push\_back(arg\_type1);

205  arg\_types.push\_back(arg\_type2);

206  arg\_types.push\_back(arg\_type3);

207  add(name,result\_type,arg\_types);

208  }

209  void function\_signatures::add(const std::string& name,

210  const expr\_type& result\_type,

211  const expr\_type& arg\_type1,

212  const expr\_type& arg\_type2,

213  const expr\_type& arg\_type3,

214  const expr\_type& arg\_type4) {

215  std::vector<expr\_type> arg\_types;

216  arg\_types.push\_back(arg\_type1);

217  arg\_types.push\_back(arg\_type2);

218  arg\_types.push\_back(arg\_type3);

219  arg\_types.push\_back(arg\_type4);

220  add(name,result\_type,arg\_types);

221  }

222  void function\_signatures::add(const std::string& name,

223  const expr\_type& result\_type,

224  const expr\_type& arg\_type1,

225  const expr\_type& arg\_type2,

226  const expr\_type& arg\_type3,

227  const expr\_type& arg\_type4,

228  const expr\_type& arg\_type5) {

229  std::vector<expr\_type> arg\_types;

230  arg\_types.push\_back(arg\_type1);

231  arg\_types.push\_back(arg\_type2);

232  arg\_types.push\_back(arg\_type3);

233  arg\_types.push\_back(arg\_type4);

234  arg\_types.push\_back(arg\_type5);

235  add(name,result\_type,arg\_types);

236  }

237  void function\_signatures::add(const std::string& name,

238  const expr\_type& result\_type,

239  const expr\_type& arg\_type1,

240  const expr\_type& arg\_type2,

241  const expr\_type& arg\_type3,

242  const expr\_type& arg\_type4,

243  const expr\_type& arg\_type5,

244  const expr\_type& arg\_type6) {

245  std::vector<expr\_type> arg\_types;

246  arg\_types.push\_back(arg\_type1);

247  arg\_types.push\_back(arg\_type2);

248  arg\_types.push\_back(arg\_type3);

249  arg\_types.push\_back(arg\_type4);

250  arg\_types.push\_back(arg\_type5);

251  arg\_types.push\_back(arg\_type6);

252  add(name,result\_type,arg\_types);

253  }

254  void function\_signatures::add(const std::string& name,

255  const expr\_type& result\_type,

256  const expr\_type& arg\_type1,

257  const expr\_type& arg\_type2,

258  const expr\_type& arg\_type3,

259  const expr\_type& arg\_type4,

260  const expr\_type& arg\_type5,

261  const expr\_type& arg\_type6,

262  const expr\_type& arg\_type7) {

263  std::vector<expr\_type> arg\_types;

264  arg\_types.push\_back(arg\_type1);

265  arg\_types.push\_back(arg\_type2);

266  arg\_types.push\_back(arg\_type3);

267  arg\_types.push\_back(arg\_type4);

268  arg\_types.push\_back(arg\_type5);

269  arg\_types.push\_back(arg\_type6);

270  arg\_types.push\_back(arg\_type7);

271  add(name,result\_type,arg\_types);

272  }

273  void function\_signatures::add\_nullary(const::std::string& name) {

274  add(name,DOUBLE\_T);

275  }

276  void function\_signatures::add\_unary(const::std::string& name) {

277  add(name,DOUBLE\_T,DOUBLE\_T);

278  }

279  void function\_signatures::add\_binary(const::std::string& name) {

280  add(name,DOUBLE\_T,DOUBLE\_T,DOUBLE\_T);

281  }

282  void function\_signatures::add\_ternary(const::std::string& name) {

283  add(name,DOUBLE\_T,DOUBLE\_T,DOUBLE\_T,DOUBLE\_T);

284  }

285  void function\_signatures::add\_quaternary(const::std::string& name) {

286  add(name,DOUBLE\_T,DOUBLE\_T,DOUBLE\_T,DOUBLE\_T,DOUBLE\_T);

287  }

288  int function\_signatures::num\_promotions(

289  const std::vector<expr\_type>& call\_args,

290  const std::vector<expr\_type>& sig\_args) {

291  if (call\_args.size() != sig\_args.size()) {

292  return -1; // failure

293  }

294  int num\_promotions = 0;

295  for (size\_t i = 0; i < call\_args.size(); ++i) {

296  if (call\_args[i] == sig\_args[i]) {

297  continue;

298  } else if (call\_args[i].is\_primitive\_int()

299  && sig\_args[i].is\_primitive\_double()) {

300  ++num\_promotions;

301  } else {

302  return -1; // failed match

303  }

304  }

305  return num\_promotions;

306  }

307  int function\_signatures::get\_signature\_matches(const std::string& name,

308  const std::vector<expr\_type>& args,

309  function\_signature\_t& signature) {

310

311  std::vector<function\_signature\_t> signatures = sigs\_map\_[name];

312  size\_t min\_promotions = std::numeric\_limits<size\_t>::max();

313  size\_t num\_matches = 0;

314  for (size\_t i = 0; i < signatures.size(); ++i) {

315  signature = signatures[i];

316  int promotions = num\_promotions(args,signature.second);

317  if (promotions < 0) continue; // no match

318  size\_t promotions\_ui = static\_cast<size\_t>(promotions);

319  if (promotions\_ui < min\_promotions) {

320  min\_promotions = promotions\_ui;

321  num\_matches = 1;

322  } else if (promotions\_ui == min\_promotions) {

323  ++num\_matches;

324  }

325  }

326  return num\_matches;

327  }

328

329  expr\_type function\_signatures::get\_result\_type(

330  const std::string& name,

331  const std::vector<expr\_type>& args,

332  std::ostream& error\_msgs) {

333  std::vector<function\_signature\_t> signatures = sigs\_map\_[name];

334  size\_t match\_index = 0;

335  size\_t min\_promotions = std::numeric\_limits<size\_t>::max();

336  size\_t num\_matches = 0;

337

338  for (size\_t i = 0; i < signatures.size(); ++i) {

339  int promotions = num\_promotions(args,signatures[i].second);

340  if (promotions < 0) continue; // no match

341  size\_t promotions\_ui = static\_cast<size\_t>(promotions);

342  if (promotions\_ui < min\_promotions) {

343  min\_promotions = promotions\_ui;

344  match\_index = i;

345  num\_matches = 1;

346  } else if (promotions\_ui == min\_promotions) {

347  ++num\_matches;

348  }

349  }

350

351  if (num\_matches == 1) {

352  return signatures[match\_index].first;

353  } else if (num\_matches == 0) {

354  error\_msgs << "no matches for function name=\"" << name << "\""

355  << std::endl;

356  } else {

357  error\_msgs << num\_matches << " matches with "

358  << min\_promotions << " integer promotions "

359  << "for function name=\"" << name << "\"" << std::endl;

360  }

361  for (size\_t i = 0; i < args.size(); ++i)

362  error\_msgs << " arg " << i << " type=" << args[i] << std::endl;

363

364  error\_msgs << "available function signatures for "

365  << name << ":" << std::endl;

366  for (size\_t i = 0; i < signatures.size(); ++i) {

367  error\_msgs << i << ". " << name << "(";

368  for (size\_t j = 0; j < signatures[i].second.size(); ++j) {

369  if (j > 0) error\_msgs << ", ";

370  error\_msgs << signatures[i].second[j];

371  }

372  error\_msgs << ") : " << signatures[i].first << std::endl;

373  }

374  return expr\_type(); // ill-formed dummy

375  }

376  function\_signatures::function\_signatures() {

377 #include <stan/gm/function\_signatures.h>

378  }

379  std::set<std::string>

380  function\_signatures::key\_set() const {

381  using std::map;

382  using std::set;

383  using std::string;

384  using std::vector;

385  set<string> result;

386  for (map<string,vector<function\_signature\_t> >::const\_iterator

387  it = sigs\_map\_.begin();

388  it != sigs\_map\_.end();

389  ++it)

390  result.insert(it->first);

391  return result;

392  }

393

394  function\_signatures\* function\_signatures::sigs\_ = 0;

395

396

397  arg\_decl::arg\_decl() { }

398  arg\_decl::arg\_decl(const expr\_type& arg\_type,

399  const std::string& name)

400  : arg\_type\_(arg\_type),

401  name\_(name) {

402  }

403  base\_var\_decl arg\_decl::base\_variable\_declaration() {

404  std::vector<expression> dims;

405  for (size\_t i = 0; i < arg\_type\_.num\_dims\_; ++i)

406  dims.push\_back(expression(int\_literal(0))); // dummy value 0

407  return base\_var\_decl(name\_,dims,arg\_type\_.base\_type\_);

408  }

409

410  function\_decl\_def::function\_decl\_def() { }

411  function\_decl\_def::function\_decl\_def(const expr\_type& return\_type,

412  const std::string& name,

413  const std::vector<arg\_decl>& arg\_decls,

414  const statement& body)

415

416  : return\_type\_(return\_type),

417  name\_(name),

418  arg\_decls\_(arg\_decls),

419  body\_(body) {

420  }

421

422  function\_decl\_defs::function\_decl\_defs() { }

423  function\_decl\_defs::function\_decl\_defs(const std::vector<function\_decl\_def>&

424  decl\_defs)

425  : decl\_defs\_(decl\_defs) {

426  }

427

428  returns\_type\_vis::returns\_type\_vis(const expr\_type& return\_type,

429  std::ostream& error\_msgs)

430  : return\_type\_(return\_type),

431  error\_msgs\_(error\_msgs) {

432  }

433  bool returns\_type\_vis::operator()(const nil& st) const {

434  error\_msgs\_ << "Expecting return, found nil statement."

435  << std::endl;

436  return false;

437  }

438  bool returns\_type\_vis::operator()(const assignment& st) const {

439  error\_msgs\_ << "Expecting return, found assignment statement."

440  << std::endl;

441  return false;

442  }

443  bool returns\_type\_vis::operator()(const sample& st) const {

444  error\_msgs\_ << "Expecting return, found sampling statement."

445  << std::endl;

446  return false;

447  }

448  bool returns\_type\_vis::operator()(const

449  increment\_log\_prob\_statement& t) const {

450  error\_msgs\_ << "Expecting return, found increment\_log\_prob statement."

451  << std::endl;

452  return false;

453  }

454  bool returns\_type\_vis::operator()(const expression& st) const {

455  error\_msgs\_ << "Expecting return, found increment\_log\_prob statement."

456  << std::endl;

457  return false;

458  }

459  bool returns\_type\_vis::operator()(const print\_statement& st) const {

460  error\_msgs\_ << "Expecting return, found print statement."

461  << std::endl;

462  return false;

463  }

464  bool returns\_type\_vis::operator()(const reject\_statement& st) const {

465  error\_msgs\_ << "Expecting return, found reject statement."

466  << std::endl;

467  return false;

468  }

469  bool returns\_type\_vis::operator()(const no\_op\_statement& st) const {

470  error\_msgs\_ << "Expecting return, found no\_op statement."

471  << std::endl;

472  return false;

473  }

474  // recursive cases

475  bool returns\_type\_vis::operator()(const statements& st) const {

476  // last statement in sequence must return type

477  if (st.statements\_.size() == 0) {

478  error\_msgs\_ << ( "Expecting return, found"

479  " statement sequence with empty body." )

480  << std::endl;

481  return false;

482  }

483  return returns\_type(return\_type\_, st.statements\_.back(), error\_msgs\_);

484  }

485  bool returns\_type\_vis::operator()(const for\_statement& st) const {

486  // body must end in appropriate return

487  return returns\_type(return\_type\_, st.statement\_, error\_msgs\_);

488  }

489  bool returns\_type\_vis::operator()(const while\_statement& st) const {

490  // body must end in appropriate return

491  return returns\_type(return\_type\_, st.body\_, error\_msgs\_);

492  }

493  bool returns\_type\_vis::operator()(const

494  conditional\_statement& st) const {

495  // all condition bodies must end in appropriate return

496  if (st.bodies\_.size() != (st.conditions\_.size() + 1)) {

497  error\_msgs\_ << ( "Expecting return, found conditional"

498  " without final else.")

499  << std::endl;

500  return false;

501  }

502  for (size\_t i = 0; i < st.bodies\_.size(); ++i)

503  if (!returns\_type(return\_type\_, st.bodies\_[i], error\_msgs\_))

504  return false;

505  return true;

506  }

507  bool returns\_type\_vis::operator()(const return\_statement& st) const {

508  // return checked for type

509  return return\_type\_ == VOID\_T

510  || is\_assignable(return\_type\_, st.return\_value\_.expression\_type(),

511  "Returned expression does not match return type",

512  error\_msgs\_);

513  }

514

515  bool returns\_type(const expr\_type& return\_type,

516  const statement& statement,

517  std::ostream& error\_msgs) {

518  if (return\_type == VOID\_T)

519  return true;

520  returns\_type\_vis vis(return\_type,error\_msgs);

521  return boost::apply\_visitor(vis,statement.statement\_);

522  }

523

524

525

526  statements::statements() { }

527  statements::statements(const std::vector<var\_decl>& local\_decl,

528  const std::vector<statement>& stmts)

529  : local\_decl\_(local\_decl),

530  statements\_(stmts) {

531  }

532

533  expr\_type expression\_type\_vis::operator()(const nil& /\*e\*/) const {

534  return expr\_type();

535  }

536  expr\_type expression\_type\_vis::operator()(const int\_literal& e) const {

537  return e.type\_;

538  }

539  expr\_type expression\_type\_vis::operator()(const double\_literal& e) const {

540  return e.type\_;

541  }

542  expr\_type expression\_type\_vis::operator()(const array\_literal& e) const {

543  return e.type\_;

544  }

545  expr\_type expression\_type\_vis::operator()(const variable& e) const {

546  return e.type\_;

547  }

548  expr\_type expression\_type\_vis::operator()(const integrate\_ode& e) const {

549  return expr\_type(DOUBLE\_T,2);

550  }

551  expr\_type expression\_type\_vis::operator()(const fun& e) const {

552  return e.type\_;

553  }

554  expr\_type expression\_type\_vis::operator()(const index\_op& e) const {

555  return e.type\_;

556  }

557  expr\_type expression\_type\_vis::operator()(const binary\_op& e) const {

558  return e.type\_;

559  }

560  expr\_type expression\_type\_vis::operator()(const unary\_op& e) const {

561  return e.type\_;

562  }

563

564  expression::expression()

565  : expr\_(nil()) {

566  }

567  expression::expression(const expression& e)

568  : expr\_(e.expr\_) {

569  }

570  expr\_type expression::expression\_type() const {

571  expression\_type\_vis vis;

572  return boost::apply\_visitor(vis,expr\_);

573  }

574  // template <typename Expr>

575  // expression::expression(const Expr& expr) : expr\_(expr) { }

576

577  expression::expression(const expression\_t& expr) : expr\_(expr) { }

578  expression::expression(const nil& expr) : expr\_(expr) { }

579  expression::expression(const int\_literal& expr) : expr\_(expr) { }

580  expression::expression(const double\_literal& expr) : expr\_(expr) { }

581  expression::expression(const array\_literal& expr) : expr\_(expr) { }

582  expression::expression(const variable& expr) : expr\_(expr) { }

583  expression::expression(const integrate\_ode& expr) : expr\_(expr) { }

584  expression::expression(const fun& expr) : expr\_(expr) { }

585  expression::expression(const index\_op& expr) : expr\_(expr) { }

586  expression::expression(const binary\_op& expr) : expr\_(expr) { }

587  expression::expression(const unary\_op& expr) : expr\_(expr) { }

588

589  printable::printable() : printable\_("") { }

590  printable::printable(const expression& expr) : printable\_(expr) { }

591  printable::printable(const std::string& msg) : printable\_(msg) { }

592  printable::printable(const printable\_t& printable)

593  : printable\_(printable) { }

594  printable::printable(const printable& printable)

595  : printable\_(printable.printable\_) { }

596

597  contains\_var::contains\_var(const variable\_map& var\_map)

598  : var\_map\_(var\_map) {

599  }

600  bool contains\_var::operator()(const nil& e) const {

601  return false;

602  }

603  bool contains\_var::operator()(const int\_literal& e) const {

604  return false;

605  }

606  bool contains\_var::operator()(const double\_literal& e) const {

607  return false;

608  }

609  bool contains\_var::operator()(const array\_literal& e) const {

610  for (size\_t i = 0; i < e.args\_.size(); ++i)

611  if (boost::apply\_visitor(\*this,e.args\_[i].expr\_))

612  return true;

613  return false;

614  }

615  bool contains\_var::operator()(const variable& e) const {

616  var\_origin vo = var\_map\_.get\_origin(e.name\_);

617  return ( vo == parameter\_origin

618  || vo == transformed\_parameter\_origin

619  || vo == local\_origin );

620  }

621  bool contains\_var::operator()(const fun& e) const {

622  for (size\_t i = 0; i < e.args\_.size(); ++i)

623  if (boost::apply\_visitor(\*this,e.args\_[i].expr\_))

624  return true;

625  return false;

626  }

627  bool contains\_var::operator()(const integrate\_ode& e) const {

628  // only init state and params may contain vars

629  return boost::apply\_visitor(\*this, e.y0\_.expr\_)

630  || boost::apply\_visitor(\*this, e.theta\_.expr\_)

631  ;

632  }

633  bool contains\_var::operator()(const index\_op& e) const {

634  return boost::apply\_visitor(\*this,e.expr\_.expr\_);

635  }

636  bool contains\_var::operator()(const binary\_op& e) const {

637  return boost::apply\_visitor(\*this,e.left.expr\_)

638  || boost::apply\_visitor(\*this,e.right.expr\_);

639  }

640  bool contains\_var::operator()(const unary\_op& e) const {

641  return boost::apply\_visitor(\*this,e.subject.expr\_);

642  }

643

644  bool is\_linear\_function(const std::string& name) {

645  return name == "add"

646  || name == "block"

647  || name == "append\_col"

648  || name == "col"

649  || name == "cols"

650  || name == "diagonal"

651  || name == "head"

652  || name == "minus"

653  || name == "negative\_infinity"

654  || name == "not\_a\_number"

655  || name == "append\_row"

656  || name == "rep\_matrix"

657  || name == "rep\_row\_vector"

658  || name == "rep\_vector"

659  || name == "row"

660  || name == "rows"

661  || name == "positive\_infinity"

662  || name == "segment"

663  || name == "subtract"

664  || name == "sum"

665  || name == "tail"

666  || name == "to\_vector"

667  || name == "to\_row\_vector"

668  || name == "to\_matrix"

669  || name == "to\_array\_1d"

670  || name == "to\_array\_2d"

671  || name == "transpose"

672  ;

673  }

674

675  bool has\_var(const expression& e,

676  const variable\_map& var\_map) {

677  contains\_var vis(var\_map);

678  return boost::apply\_visitor(vis,e.expr\_);

679  }

680

681  contains\_nonparam\_var::contains\_nonparam\_var(const variable\_map& var\_map)

682  : var\_map\_(var\_map) {

683  }

684  bool contains\_nonparam\_var::operator()(const nil& e) const {

685  return false;

686  }

687  bool contains\_nonparam\_var::operator()(const int\_literal& e) const {

688  return false;

689  }

690  bool contains\_nonparam\_var::operator()(const double\_literal& e) const {

691  return false;

692  }

693  bool contains\_nonparam\_var::operator()(const array\_literal& e) const {

694  for (size\_t i = 0; i < e.args\_.size(); ++i)

695  if (boost::apply\_visitor(\*this,e.args\_[i].expr\_))

696  return true;

697  return false;

698  }

699  bool contains\_nonparam\_var::operator()(const variable& e) const {

700  var\_origin vo = var\_map\_.get\_origin(e.name\_);

701  return ( vo == transformed\_parameter\_origin

702  || vo == local\_origin );

703  }

704  bool contains\_nonparam\_var::operator()(const integrate\_ode& e) const {

705  // if any vars, return true because integration will be nonlinear

706  return boost::apply\_visitor(\*this, e.y0\_.expr\_)

707  || boost::apply\_visitor(\*this, e.theta\_.expr\_)

708  ;

709  }

710  bool contains\_nonparam\_var::operator()(const fun& e) const {

711  // any function applied to non-linearly transformed var

712  for (size\_t i = 0; i < e.args\_.size(); ++i)

713  if (boost::apply\_visitor(\*this,e.args\_[i].expr\_))

714  return true;

715  // non-linear function applied to var

716  if (!is\_linear\_function(e.name\_)) {

717  for (size\_t i = 0; i < e.args\_.size(); ++i)

718  if (has\_var(e.args\_[i],var\_map\_))

719  return true;

720  }

721  return false;

722  }

723  bool contains\_nonparam\_var::operator()(const index\_op& e) const {

724  return boost::apply\_visitor(\*this,e.expr\_.expr\_);

725  }

726  bool contains\_nonparam\_var::operator()(const binary\_op& e) const {

727  if (e.op == "||"

728  || e.op == "&&"

729  || e.op == "=="

730  || e.op == "!="

731  || e.op == "<"

732  || e.op == "<="

733  || e.op == ">"

734  || e.op == ">=")

735  return true;

736  if (has\_non\_param\_var(e.left,var\_map\_)

737  || has\_non\_param\_var(e.right,var\_map\_))

738  return true;

739  if (e.op == "\*" || e.op == "/")

740  return has\_var(e.left,var\_map\_) && has\_var(e.right,var\_map\_);

741  return false;

742  }

743  bool contains\_nonparam\_var::operator()(const unary\_op& e) const {

744  // only negation, which is linear, so recurse

745  return has\_non\_param\_var(e.subject,var\_map\_);

746  }

747

748  bool has\_non\_param\_var(const expression& e,

749  const variable\_map& var\_map) {

750  contains\_nonparam\_var vis(var\_map);

751  return boost::apply\_visitor(vis,e.expr\_);

752  }

753

754  bool is\_nil\_op::operator()(const nil& /\*x\*/) const { return true; }

755  bool is\_nil\_op::operator()(const int\_literal& /\*x\*/) const { return false; }

756  bool is\_nil\_op::operator()(const double\_literal& /\* x \*/) const { return false; }

757  bool is\_nil\_op::operator()(const array\_literal& /\* x \*/) const { return false; }

758  bool is\_nil\_op::operator()(const variable& /\* x \*/) const { return false; }

759  bool is\_nil\_op::operator()(const integrate\_ode& /\* x \*/) const { return false; }

760  bool is\_nil\_op::operator()(const fun& /\* x \*/) const { return false; }

761  bool is\_nil\_op::operator()(const index\_op& /\* x \*/) const { return false; }

762  bool is\_nil\_op::operator()(const binary\_op& /\* x \*/) const { return false; }

763  bool is\_nil\_op::operator()(const unary\_op& /\* x \*/) const { return false; }

764

765  // template <typename T>

766  // bool is\_nil\_op::operator()(const T& /\* x \*/) const { return false; }

767

768  bool is\_nil(const expression& e) {

769  is\_nil\_op ino;

770  return boost::apply\_visitor(ino,e.expr\_);

771  }

772

773  variable\_dims::variable\_dims() { } // req for FUSION\_ADAPT

774  variable\_dims::variable\_dims(std::string const& name,

775  std::vector<expression> const& dims)

776  : name\_(name),

777  dims\_(dims) {

778  }

779

780

781  int\_literal::int\_literal()

782  : type\_(INT\_T) {

783  }

784  int\_literal::int\_literal(int val)

785  : val\_(val),

786  type\_(INT\_T) {

787  }

788  int\_literal::int\_literal(const int\_literal& il)

789  : val\_(il.val\_),

790  type\_(il.type\_) {

791  }

792  int\_literal& int\_literal::operator=(const int\_literal& il) {

793  val\_ = il.val\_;

794  type\_ = il.type\_;

795  return \*this;

796  }

797

798

799  double\_literal::double\_literal()

800  : type\_(DOUBLE\_T,0U) {

801  }

802  double\_literal::double\_literal(double val)

803  : val\_(val),

804  type\_(DOUBLE\_T,0U) {

805  }

806  double\_literal& double\_literal::operator=(const double\_literal& dl) {

807  val\_ = dl.val\_;

808  type\_ = dl.type\_;

809  return \*this;

810  }

811

812

813  array\_literal::array\_literal()

814  : args\_(),

815  type\_(DOUBLE\_T,1U) {

816  }

817  array\_literal::array\_literal(const std::vector<expression>& args)

818  : args\_(args),

819  type\_() { // ill-formed w/o help

820  }

821  array\_literal& array\_literal::operator=(const array\_literal& al) {

822  args\_ = al.args\_;

823  type\_ = al.type\_;

824  return \*this;

825  }

826

827  variable::variable() { }

828  variable::variable(std::string name) : name\_(name) { }

829  void variable::set\_type(const base\_expr\_type& base\_type,

830  size\_t num\_dims) {

831  type\_ = expr\_type(base\_type, num\_dims);

832  }

833

834

835  integrate\_ode::integrate\_ode() { }

836  integrate\_ode::integrate\_ode(const std::string& system\_function\_name,

837  const expression& y0,

838  const expression& t0,

839  const expression& ts,

840  const expression& theta,

841  const expression& x,

842  const expression& x\_int)

843  : system\_function\_name\_(system\_function\_name),

844  y0\_(y0),

845  t0\_(t0),

846  ts\_(ts),

847  theta\_(theta),

848  x\_(x),

849  x\_int\_(x\_int) {

850  }

851

852

853  fun::fun() { }

854  fun::fun(std::string const& name,

855  std::vector<expression> const& args)

856  : name\_(name),

857  args\_(args) {

858  infer\_type();

859  }

860  void fun::infer\_type() {

861  // FIXME: remove this useless function and any calls to it

862  }

863

864

865  size\_t total\_dims(const std::vector<std::vector<expression> >& dimss) {

866  size\_t total = 0U;

867  for (size\_t i = 0; i < dimss.size(); ++i)

868  total += dimss[i].size();

869  return total;

870  }

871

872

873  expr\_type infer\_type\_indexing(const base\_expr\_type& expr\_base\_type,

874  size\_t num\_expr\_dims,

875  size\_t num\_index\_dims) {

876  if (num\_index\_dims <= num\_expr\_dims)

877  return expr\_type(expr\_base\_type,num\_expr\_dims - num\_index\_dims);

878  if (num\_index\_dims == (num\_expr\_dims + 1)) {

879  if (expr\_base\_type == VECTOR\_T || expr\_base\_type == ROW\_VECTOR\_T)

880  return expr\_type(DOUBLE\_T,0U);

881  if (expr\_base\_type == MATRIX\_T)

882  return expr\_type(ROW\_VECTOR\_T,0U);

883  }

884  if (num\_index\_dims == (num\_expr\_dims + 2))

885  if (expr\_base\_type == MATRIX\_T)

886  return expr\_type(DOUBLE\_T,0U);

887

888  // error condition, result expr\_type has is\_ill\_formed() = true

889  return expr\_type();

890  }

891

892  expr\_type infer\_type\_indexing(const expression& expr,

893  size\_t num\_index\_dims) {

894  return infer\_type\_indexing(expr.expression\_type().base\_type\_,

895  expr.expression\_type().num\_dims(),

896  num\_index\_dims);

897  }

898

899

900  index\_op::index\_op() { }

901  index\_op::index\_op(const expression& expr,

902  const std::vector<std::vector<expression> >& dimss)

903  : expr\_(expr),

904  dimss\_(dimss) {

905  infer\_type();

906  }

907  void index\_op::infer\_type() {

908  type\_ = infer\_type\_indexing(expr\_,total\_dims(dimss\_));

909  }

910

911  binary\_op::binary\_op() { }

912  binary\_op::binary\_op(const expression& left,

913  const std::string& op,

914  const expression& right)

915  : op(op),

916  left(left),

917  right(right),

918  type\_(promote\_primitive(left.expression\_type(),

919  right.expression\_type())) {

920  }

921

922

923  unary\_op::unary\_op(char op,

924  expression const& subject)

925  : op(op),

926  subject(subject),

927  type\_(promote\_primitive(subject.expression\_type())) {

928  }

929

930

931  range::range() { }

932  range::range(expression const& low,

933  expression const& high)

934  : low\_(low),

935  high\_(high) {

936  }

937  bool range::has\_low() const {

938  return !is\_nil(low\_.expr\_);

939  }

940  bool range::has\_high() const {

941  return !is\_nil(high\_.expr\_);

942  }

943

944  void print\_var\_origin(std::ostream& o, const var\_origin& vo) {

945  if (vo == model\_name\_origin)

946  o << "model name";

947  else if (vo == data\_origin)

948  o << "data";

949  else if (vo == transformed\_data\_origin)

950  o << "transformed data";

951  else if (vo == parameter\_origin)

952  o << "parameter";

953  else if (vo == transformed\_parameter\_origin)

954  o << "transformed parameter";

955  else if (vo == derived\_origin)

956  o << "generated quantities";

957  else if (vo == local\_origin)

958  o << "local";

959  else if (vo == function\_argument\_origin)

960  o << "function argument";

961  else if (vo == function\_argument\_origin\_lp)

962  o << "function argument '\_lp' suffixed";

963  else if (vo == function\_argument\_origin\_rng)

964  o << "function argument '\_rng' suffixed";

965  else if (vo == void\_function\_argument\_origin)

966  o << "void function argument";

967  else if (vo == void\_function\_argument\_origin\_lp)

968  o << "void function argument '\_lp' suffixed";

969  else if (vo == void\_function\_argument\_origin\_rng)

970  o << "void function argument '\_rng' suffixed";

971  else

972  o << "UNKNOWN ORIGIN=" << vo;

973  }

974

975

976  base\_var\_decl::base\_var\_decl() { }

977  base\_var\_decl::base\_var\_decl(const base\_expr\_type& base\_type)

978  : base\_type\_(base\_type) {

979  }

980  base\_var\_decl::base\_var\_decl(const std::string& name,

981  const std::vector<expression>& dims,

982  const base\_expr\_type& base\_type)

983  : name\_(name),

984  dims\_(dims),

985  base\_type\_(base\_type) {

986  }

987

988  bool variable\_map::exists(const std::string& name) const {

989  return map\_.find(name) != map\_.end();

990  }

991  base\_var\_decl variable\_map::get(const std::string& name) const {

992  if (!exists(name))

993  throw std::invalid\_argument("variable does not exist");

994  return map\_.find(name)->second.first;

995  }

996  base\_expr\_type variable\_map::get\_base\_type(const std::string& name) const {

997  return get(name).base\_type\_;

998  }

999  size\_t variable\_map::get\_num\_dims(const std::string& name) const {

1000  return get(name).dims\_.size();

1001  }

1002  var\_origin variable\_map::get\_origin(const std::string& name) const {

1003  if (!exists(name))

1004  throw std::invalid\_argument("variable does not exist");

1005  return map\_.find(name)->second.second;

1006  }

1007  void variable\_map::add(const std::string& name,

1008  const base\_var\_decl& base\_decl,

1009  const var\_origin& vo) {

1010  map\_[name] = range\_t(base\_decl,vo);

1011  }

1012  void variable\_map::remove(const std::string& name) {

1013  map\_.erase(name);

1014  }

1015

1016  int\_var\_decl::int\_var\_decl()

1017  : base\_var\_decl(INT\_T)

1018  { }

1019

1020  int\_var\_decl::int\_var\_decl(range const& range,

1021  std::string const& name,

1022  std::vector<expression> const& dims)

1023  : base\_var\_decl(name,dims,INT\_T),

1024  range\_(range)

1025  { }

1026

1027

1028

1029  double\_var\_decl::double\_var\_decl()

1030  : base\_var\_decl(DOUBLE\_T)

1031  { }

1032

1033  double\_var\_decl::double\_var\_decl(range const& range,

1034  std::string const& name,

1035  std::vector<expression> const& dims)

1036  : base\_var\_decl(name,dims,DOUBLE\_T),

1037  range\_(range)

1038  { }

1039

1040  unit\_vector\_var\_decl::unit\_vector\_var\_decl()

1041  : base\_var\_decl(VECTOR\_T)

1042  { }

1043

1044  unit\_vector\_var\_decl::unit\_vector\_var\_decl(expression const& K,

1045  std::string const& name,

1046  std::vector<expression> const& dims)

1047  : base\_var\_decl(name,dims,VECTOR\_T),

1048  K\_(K)

1049  { }

1050

1051  simplex\_var\_decl::simplex\_var\_decl()

1052  : base\_var\_decl(VECTOR\_T)

1053  { }

1054

1055  simplex\_var\_decl::simplex\_var\_decl(expression const& K,

1056  std::string const& name,

1057  std::vector<expression> const& dims)

1058  : base\_var\_decl(name,dims,VECTOR\_T),

1059  K\_(K)

1060  { }

1061

1062  ordered\_var\_decl::ordered\_var\_decl()

1063  : base\_var\_decl(VECTOR\_T)

1064  { }

1065

1066  ordered\_var\_decl::ordered\_var\_decl(expression const& K,

1067  std::string const& name,

1068  std::vector<expression> const& dims)

1069  : base\_var\_decl(name,dims,VECTOR\_T),

1070  K\_(K) {

1071  }

1072

1073  positive\_ordered\_var\_decl::positive\_ordered\_var\_decl()

1074  : base\_var\_decl(VECTOR\_T)

1075  { }

1076

1077  positive\_ordered\_var\_decl::positive\_ordered\_var\_decl(expression const& K,

1078  std::string const& name,

1079  std::vector<expression> const& dims)

1080  : base\_var\_decl(name,dims,VECTOR\_T),

1081  K\_(K) {

1082  }

1083

1084  vector\_var\_decl::vector\_var\_decl() : base\_var\_decl(VECTOR\_T) { }

1085

1086  vector\_var\_decl::vector\_var\_decl(range const& range,

1087  expression const& M,

1088  std::string const& name,

1089  std::vector<expression> const& dims)

1090  : base\_var\_decl(name,dims,VECTOR\_T),

1091  range\_(range),

1092  M\_(M) {

1093  }

1094

1095  row\_vector\_var\_decl::row\_vector\_var\_decl() : base\_var\_decl(ROW\_VECTOR\_T) { }

1096  row\_vector\_var\_decl::row\_vector\_var\_decl(range const& range,

1097  expression const& N,

1098  std::string const& name,

1099  std::vector<expression> const& dims)

1100  : base\_var\_decl(name,dims,ROW\_VECTOR\_T),

1101  range\_(range),

1102  N\_(N) {

1103  }

1104

1105  matrix\_var\_decl::matrix\_var\_decl() : base\_var\_decl(MATRIX\_T) { }

1106  matrix\_var\_decl::matrix\_var\_decl(range const& range,

1107  expression const& M,

1108  expression const& N,

1109  std::string const& name,

1110  std::vector<expression> const& dims)

1111  : base\_var\_decl(name,dims,MATRIX\_T),

1112  range\_(range),

1113  M\_(M),

1114  N\_(N) {

1115  }

1116

1117

1118  cholesky\_factor\_var\_decl::cholesky\_factor\_var\_decl()

1119  : base\_var\_decl(MATRIX\_T) {

1120  }

1121  cholesky\_factor\_var\_decl::cholesky\_factor\_var\_decl(expression const& M,

1122  expression const& N,

1123  std::string const& name,

1124  std::vector<expression> const& dims)

1125  : base\_var\_decl(name,dims,MATRIX\_T),

1126  M\_(M),

1127  N\_(N) {

1128  }

1129

1130  cholesky\_corr\_var\_decl::cholesky\_corr\_var\_decl()

1131  : base\_var\_decl(MATRIX\_T) {

1132  }

1133  cholesky\_corr\_var\_decl::cholesky\_corr\_var\_decl(expression const& K,

1134  std::string const& name,

1135  std::vector<expression> const& dims)

1136  : base\_var\_decl(name,dims,MATRIX\_T),

1137  K\_(K) {

1138  }

1139

1140  cov\_matrix\_var\_decl::cov\_matrix\_var\_decl() : base\_var\_decl(MATRIX\_T) {

1141  }

1142  cov\_matrix\_var\_decl::cov\_matrix\_var\_decl(expression const& K,

1143  std::string const& name,

1144  std::vector<expression> const& dims)

1145  : base\_var\_decl(name,dims,MATRIX\_T),

1146  K\_(K) {

1147  }

1148

1149  corr\_matrix\_var\_decl::corr\_matrix\_var\_decl() : base\_var\_decl(MATRIX\_T) { }

1150  corr\_matrix\_var\_decl::corr\_matrix\_var\_decl(expression const& K,

1151  std::string const& name,

1152  std::vector<expression> const& dims)

1153  : base\_var\_decl(name,dims,MATRIX\_T),

1154  K\_(K) {

1155  }

1156

1157

1158

1159

1160  name\_vis::name\_vis() { }

1161  std::string name\_vis::operator()(const nil& /\* x \*/) const {

1162  return ""; // fail if arises

1163  }

1164  std::string name\_vis::operator()(const int\_var\_decl& x) const {

1165  return x.name\_;

1166  }

1167  std::string name\_vis::operator()(const double\_var\_decl& x) const {

1168  return x.name\_;

1169  }

1170  std::string name\_vis::operator()(const vector\_var\_decl& x) const {

1171  return x.name\_;

1172  }

1173  std::string name\_vis::operator()(const row\_vector\_var\_decl& x) const {

1174  return x.name\_;

1175  }

1176  std::string name\_vis::operator()(const matrix\_var\_decl& x) const {

1177  return x.name\_;

1178  }

1179  std::string name\_vis::operator()(const unit\_vector\_var\_decl& x) const {

1180  return x.name\_;

1181  }

1182  std::string name\_vis::operator()(const simplex\_var\_decl& x) const {

1183  return x.name\_;

1184  }

1185  std::string name\_vis::operator()(const ordered\_var\_decl& x) const {

1186  return x.name\_;

1187  }

1188  std::string name\_vis::operator()(const positive\_ordered\_var\_decl& x) const {

1189  return x.name\_;

1190  }

1191  std::string name\_vis::operator()(const cholesky\_factor\_var\_decl& x) const {

1192  return x.name\_;

1193  }

1194  std::string name\_vis::operator()(const cholesky\_corr\_var\_decl& x) const {

1195  return x.name\_;

1196  }

1197  std::string name\_vis::operator()(const cov\_matrix\_var\_decl& x) const {

1198  return x.name\_;

1199  }

1200  std::string name\_vis::operator()(const corr\_matrix\_var\_decl& x) const {

1201  return x.name\_;

1202  }

1203

1204

1205  // can't template out in .cpp file

1206

1207  var\_decl::var\_decl(const var\_decl\_t& decl) : decl\_(decl) { }

1208  var\_decl::var\_decl() : decl\_(nil()) { }

1209  var\_decl::var\_decl(const nil& decl) : decl\_(decl) { }

1210  var\_decl::var\_decl(const int\_var\_decl& decl) : decl\_(decl) { }

1211  var\_decl::var\_decl(const double\_var\_decl& decl) : decl\_(decl) { }

1212  var\_decl::var\_decl(const vector\_var\_decl& decl) : decl\_(decl) { }

1213  var\_decl::var\_decl(const row\_vector\_var\_decl& decl) : decl\_(decl) { }

1214  var\_decl::var\_decl(const matrix\_var\_decl& decl) : decl\_(decl) { }

1215  var\_decl::var\_decl(const unit\_vector\_var\_decl& decl) : decl\_(decl) { }

1216  var\_decl::var\_decl(const simplex\_var\_decl& decl) : decl\_(decl) { }

1217  var\_decl::var\_decl(const ordered\_var\_decl& decl) : decl\_(decl) { }

1218  var\_decl::var\_decl(const positive\_ordered\_var\_decl& decl) : decl\_(decl) { }

1219  var\_decl::var\_decl(const cholesky\_factor\_var\_decl& decl) : decl\_(decl) { }

1220  var\_decl::var\_decl(const cholesky\_corr\_var\_decl& decl) : decl\_(decl) { }

1221  var\_decl::var\_decl(const cov\_matrix\_var\_decl& decl) : decl\_(decl) { }

1222  var\_decl::var\_decl(const corr\_matrix\_var\_decl& decl) : decl\_(decl) { }

1223

1224  std::string var\_decl::name() const {

1225  return boost::apply\_visitor(name\_vis(),decl\_);

1226  }

1227

1228  statement::statement() : statement\_(nil()) { }

1229

1230  statement::statement(const statement\_t& st) : statement\_(st) { }

1231  statement::statement(const nil& st) : statement\_(st) { }

1232  statement::statement(const assignment& st) : statement\_(st) { }

1233  statement::statement(const sample& st) : statement\_(st) { }

1234  statement::statement(const increment\_log\_prob\_statement& st) : statement\_(st) { }

1235  statement::statement(const statements& st) : statement\_(st) { }

1236  statement::statement(const expression& st) : statement\_(st) { }

1237  statement::statement(const for\_statement& st) : statement\_(st) { }

1238  statement::statement(const while\_statement& st) : statement\_(st) { }

1239  statement::statement(const conditional\_statement& st) : statement\_(st) { }

1240  statement::statement(const print\_statement& st) : statement\_(st) { }

1241  statement::statement(const reject\_statement& st) : statement\_(st) { }

1242  statement::statement(const return\_statement& st) : statement\_(st) { }

1243  statement::statement(const no\_op\_statement& st) : statement\_(st) { }

1244

1245

1246  bool is\_no\_op\_statement\_vis::operator()(const nil& st) const {

1247  return false;

1248  }

1249  bool is\_no\_op\_statement\_vis::operator()(const assignment& st) const {

1250  return false;

1251  }

1252  bool is\_no\_op\_statement\_vis::operator()(const sample& st) const {

1253  return false;

1254  }

1255  bool is\_no\_op\_statement\_vis::operator()(const increment\_log\_prob\_statement& t) const {

1256  return false;

1257  }

1258  bool is\_no\_op\_statement\_vis::operator()(const expression& st) const {

1259  return false;

1260  }

1261  bool is\_no\_op\_statement\_vis::operator()(const statements& st) const {

1262  return false;

1263  }

1264  bool is\_no\_op\_statement\_vis::operator()(const for\_statement& st) const {

1265  return false;

1266  }

1267  bool is\_no\_op\_statement\_vis::operator()(const conditional\_statement& st) const {

1268  return false;

1269  }

1270  bool is\_no\_op\_statement\_vis::operator()(const while\_statement& st) const {

1271  return false;

1272  }

1273  bool is\_no\_op\_statement\_vis::operator()(const print\_statement& st) const {

1274  return false;

1275  }

1276  bool is\_no\_op\_statement\_vis::operator()(const reject\_statement& st) const {

1277  return false;

1278  }

1279  bool is\_no\_op\_statement\_vis::operator()(const no\_op\_statement& st) const {

1280  return true;

1281  }

1282  bool is\_no\_op\_statement\_vis::operator()(const return\_statement& st) const {

1283  return false;

1284  }

1285

1286  bool statement::is\_no\_op\_statement() const {

1287  is\_no\_op\_statement\_vis vis;

1288  return boost::apply\_visitor(vis,statement\_);

1289  }

1290

1291  increment\_log\_prob\_statement::increment\_log\_prob\_statement() {

1292  }

1293  increment\_log\_prob\_statement::increment\_log\_prob\_statement(const expression& log\_prob)

1294  : log\_prob\_(log\_prob) {

1295  }

1296

1297  for\_statement::for\_statement() {

1298  }

1299  for\_statement::for\_statement(std::string& variable,

1300  range& range,

1301  statement& stmt)

1302  : variable\_(variable),

1303  range\_(range),

1304  statement\_(stmt) {

1305  }

1306

1307  while\_statement::while\_statement() {

1308  }

1309  while\_statement::while\_statement(const expression& condition,

1310  const statement& body)

1311  : condition\_(condition),

1312  body\_(body) {

1313  }

1314

1315  conditional\_statement::conditional\_statement() {

1316  }

1317  conditional\_statement

1318  ::conditional\_statement(const std::vector<expression>& conditions,

1319  const std::vector<statement>& bodies)

1320  : conditions\_(conditions),

1321  bodies\_(bodies) {

1322  }

1323

1324  return\_statement::return\_statement() { }

1325  return\_statement::return\_statement(const expression& expr)

1326  : return\_value\_(expr) {

1327  }

1328

1329  print\_statement::print\_statement() { }

1330

1331  print\_statement::print\_statement(const std::vector<printable>& printables)

1332  : printables\_(printables) {

1333  }

1334

1335  reject\_statement::reject\_statement() { }

1336

1337  reject\_statement::reject\_statement(const std::vector<printable>& printables)

1338  : printables\_(printables) {

1339  }

1340

1341  program::program() { }

1342  program::program(const std::vector<function\_decl\_def>& function\_decl\_defs,

1343  const std::vector<var\_decl>& data\_decl,

1344  const std::pair<std::vector<var\_decl>,

1345  std::vector<statement> >& derived\_data\_decl,

1346  const std::vector<var\_decl>& parameter\_decl,

1347  const std::pair<std::vector<var\_decl>,

1348  std::vector<statement> >& derived\_decl,

1349  const statement& st,

1350  const std::pair<std::vector<var\_decl>,

1351  std::vector<statement> >& generated\_decl)

1352  : function\_decl\_defs\_(function\_decl\_defs),

1353  data\_decl\_(data\_decl),

1354  derived\_data\_decl\_(derived\_data\_decl),

1355  parameter\_decl\_(parameter\_decl),

1356  derived\_decl\_(derived\_decl),

1357  statement\_(st),

1358  generated\_decl\_(generated\_decl) {

1359  }

1360

1361  sample::sample() {

1362  }

1363  sample::sample(expression& e,

1364  distribution& dist)

1365  : expr\_(e),

1366  dist\_(dist) {

1367  }

1368  bool sample::is\_ill\_formed() const {

1369  return expr\_.expression\_type().is\_ill\_formed()

1370  || ( truncation\_.has\_low()

1371  && expr\_.expression\_type()

1372  != truncation\_.low\_.expression\_type() )

1373  || ( truncation\_.has\_high()

1374  && expr\_.expression\_type()

1375  != truncation\_.high\_.expression\_type() );

1376  }

1377

1378  assignment::assignment() {

1379  }

1380  assignment::assignment(variable\_dims& var\_dims,

1381  expression& expr)

1382  : var\_dims\_(var\_dims),

1383  expr\_(expr) {

1384  }

1385

1386  expression& expression::operator+=(const expression& rhs) {

1387  expr\_ = binary\_op(expr\_, "+", rhs);

1388  return \*this;

1389  }

1390

1391  expression& expression::operator-=(const expression& rhs) {

1392  expr\_ = binary\_op(expr\_, "-", rhs);

1393  return \*this;

1394  }

1395

1396  expression& expression::operator\*=(expression const& rhs) {

1397  expr\_ = binary\_op(expr\_, "\*", rhs);

1398  return \*this;

1399  }

1400

1401  expression& expression::operator/=(expression const& rhs) {

1402  expr\_ = binary\_op(expr\_, "/", rhs);

1403  return \*this;

1404  }

1405

1406  bool has\_rng\_suffix(const std::string& s) {

1407  int n = s.size();

1408  return n > 4

1409  && s[n-1] == 'g'

1410  && s[n-2] == 'n'

1411  && s[n-3] == 'r'

1412  && s[n-4] == '\_';

1413  }

1414

1415  bool has\_lp\_suffix(const std::string& s) {

1416  int n = s.size();

1417  return n > 3

1418  && s[n-1] == 'p'

1419  && s[n-2] == 'l'

1420  && s[n-3] == '\_';

1421  }

1422

1423  bool is\_user\_defined(const std::string& name,

1424  const std::vector<expression>& args) {

1425  std::vector<expr\_type> arg\_types;

1426  for (size\_t i = 0; i < args.size(); ++i)

1427  arg\_types.push\_back(args[i].expression\_type());

1428  function\_signature\_t sig;

1429  int matches

1430  = function\_signatures::instance()

1431  .get\_signature\_matches(name,arg\_types,sig);

1432  if (matches != 1)

1433  return false; // reall shouldn't come up; throw instead?

1434  std::pair<std::string, function\_signature\_t>

1435  name\_sig(name, sig);

1436  return function\_signatures::instance().is\_user\_defined(name\_sig);

1437  }

1438

1439  bool is\_user\_defined\_prob\_function(const std::string& name,

1440  const expression& variate,

1441  const std::vector<expression>& params) {

1442  std::vector<expression> variate\_params;

1443  variate\_params.push\_back(variate);

1444  for (size\_t i = 0; i < params.size(); ++i)

1445  variate\_params.push\_back(params[i]);

1446  return is\_user\_defined(name,variate\_params);

1447  }

1448

1449  bool is\_user\_defined(const fun& fx) {

1450  return is\_user\_defined(fx.name\_, fx.args\_);

1451  }

1452

1453  bool is\_assignable(const expr\_type& l\_type,

1454  const expr\_type& r\_type,

1455  const std::string& failure\_message,

1456  std::ostream& error\_msgs) {

1457  bool assignable = true;

1458  if (l\_type.num\_dims\_ != r\_type.num\_dims\_) {

1459  assignable = false;

1460  error\_msgs << "Mismatched array dimensions.";

1461  }

1462  if (l\_type.base\_type\_ != r\_type.base\_type\_

1463  && (! (l\_type.base\_type\_ == DOUBLE\_T && r\_type.base\_type\_ == INT\_T))) {

1464  assignable = false;

1465  error\_msgs << "Base type mismatch. ";

1466  }

1467  if (!assignable)

1468  error\_msgs << failure\_message

1469  << std::endl

1470  << " LHS type = " << l\_type

1471  << "; RHS type = " << r\_type

1472  << std::endl;

1473  return assignable;

1474  }

1475

1476

1477  bool ends\_with(const std::string& suffix,

1478  const std::string& s) {

1479  size\_t idx = s.rfind(suffix);

1480  return idx != std::string::npos

1481  && idx == (s.size() - suffix.size());

1482  }

1483

1484  }

1485 }

1486

1487

1488 #endif

stan::gm::var\_decl::var\_decl

var\_decl()

**Definition:** ast\_def.cpp:1208

stan::gm::expr\_type::operator==

bool operator==(const expr\_type &et) const

**Definition:** ast\_def.cpp:66

stan::gm::nil

Placeholder struct for boost::variant default ctors.

**Definition:** ast.hpp:18

stan::gm::expression::operator/=

expression & operator/=(const expression &rhs)

**Definition:** ast\_def.cpp:1401

stan::gm::variable\_dims

**Definition:** ast.hpp:288

stan::gm::statement

**Definition:** ast.hpp:634

stan::gm::function\_signatures::is\_user\_defined

bool is\_user\_defined(const std::pair< std::string, function\_signature\_t > &name\_sig)

**Definition:** ast\_def.cpp:157

stan::gm::statement::is\_no\_op\_statement

bool is\_no\_op\_statement() const

**Definition:** ast\_def.cpp:1286

stan::gm::function\_signatures::add\_unary

void add\_unary(const ::std::string &name)

**Definition:** ast\_def.cpp:276

stan::gm::corr\_matrix\_var\_decl

**Definition:** ast.hpp:563

stan::gm::ends\_with

bool ends\_with(const std::string &suffix, const std::string &s)

**Definition:** ast\_def.cpp:1477

stan::gm::cov\_matrix\_var\_decl::cov\_matrix\_var\_decl

cov\_matrix\_var\_decl()

**Definition:** ast\_def.cpp:1140

N\_

int N\_

**Definition:** mdivide\_left.hpp:20

stan::gm::conditional\_statement::conditions\_

std::vector< expression > conditions\_

**Definition:** ast.hpp:730

stan::gm::fun::name\_

std::string name\_

**Definition:** ast.hpp:351

stan::gm::function\_signatures::add

void add(const std::string &name, const expr\_type &result\_type, const std::vector< expr\_type > &arg\_types)

**Definition:** ast\_def.cpp:171

stan::gm::binary\_op::right

expression right

**Definition:** ast.hpp:385

stan::gm::expr\_type::base\_type\_

base\_expr\_type base\_type\_

**Definition:** ast.hpp:75

stan::gm::data\_origin

const int data\_origin

**Definition:** ast.hpp:413

stan::gm::function\_signatures::add\_nullary

void add\_nullary(const ::std::string &name)

**Definition:** ast\_def.cpp:273

stan::gm::increment\_log\_prob\_statement::increment\_log\_prob\_statement

increment\_log\_prob\_statement()

**Definition:** ast\_def.cpp:1291

stan::gm::double\_var\_decl

**Definition:** ast.hpp:464

stan::gm::parameter\_origin

const int parameter\_origin

**Definition:** ast.hpp:415

stan::gm::print\_statement::print\_statement

print\_statement()

**Definition:** ast\_def.cpp:1329

stan::gm::variable\_map::add

void add(const std::string &name, const base\_var\_decl &base\_decl, const var\_origin &vo)

**Definition:** ast\_def.cpp:1007

stan::gm::fun::type\_

expr\_type type\_

**Definition:** ast.hpp:353

stan::gm::is\_user\_defined\_prob\_function

bool is\_user\_defined\_prob\_function(const std::string &name, const expression &variate, const std::vector< expression > &params)

**Definition:** ast\_def.cpp:1439

stan::gm::function\_signatures::is\_defined

bool is\_defined(const std::string &name, const function\_signature\_t &sig)

**Definition:** ast\_def.cpp:163

stan::gm::DOUBLE\_T

const int DOUBLE\_T

**Definition:** ast.hpp:66

stan::gm::arg\_decl::name\_

std::string name\_

**Definition:** ast.hpp:769

stan::gm::row\_vector\_var\_decl

**Definition:** ast.hpp:514

stan::gm::unary\_op

**Definition:** ast.hpp:393

stan::gm::function\_signatures::instance

static function\_signatures & instance()

**Definition:** ast\_def.cpp:143

stan::gm::integrate\_ode::y0\_

expression y0\_

**Definition:** ast.hpp:334

stan::gm::fun::infer\_type

void infer\_type()

**Definition:** ast\_def.cpp:860

stan::gm::array\_literal

**Definition:** ast.hpp:315

stan::gm::local\_origin

const int local\_origin

**Definition:** ast.hpp:418

stan::gm::distribution

**Definition:** ast.hpp:194

stan::gm::statements

**Definition:** ast.hpp:185

stan::gm::range

**Definition:** ast.hpp:401

stan::gm::function\_decl\_defs::function\_decl\_defs

function\_decl\_defs()

**Definition:** ast\_def.cpp:422

stan::gm::variable\_map::map\_

std::map< std::string, range\_t > map\_

**Definition:** ast.hpp:452

stan::gm::printable::printable\_t

boost::variant< boost::recursive\_wrapper< std::string >, boost::recursive\_wrapper< expression > > printable\_t

**Definition:** ast.hpp:259

stan::gm::unary\_op::type\_

expr\_type type\_

**Definition:** ast.hpp:396

stan::gm::conditional\_statement

**Definition:** ast.hpp:729

stan::gm::assignment

**Definition:** ast.hpp:828

stan::gm::ordered\_var\_decl

**Definition:** ast.hpp:488

stan::gm::index\_op::dimss\_

std::vector< std::vector< expression > > dimss\_

**Definition:** ast.hpp:372

stan::gm::expr\_type::operator>=

bool operator>=(const expr\_type &et) const

**Definition:** ast\_def.cpp:88

stan::gm::variable::variable

variable()

**Definition:** ast\_def.cpp:827

stan::gm::expr\_type::is\_primitive\_int

bool is\_primitive\_int() const

**Definition:** ast\_def.cpp:97

stan::gm::binary\_op::type\_

expr\_type type\_

**Definition:** ast.hpp:386

stan::gm::function\_signatures::set\_user\_defined

void set\_user\_defined(const std::pair< std::string, function\_signature\_t > &name\_sig)

**Definition:** ast\_def.cpp:150

stan::gm::int\_literal

**Definition:** ast.hpp:297

stan::gm::range::has\_low

bool has\_low() const

**Definition:** ast\_def.cpp:937

stan::gm::range::high\_

expression high\_

**Definition:** ast.hpp:403

stan::gm::expr\_type::is\_primitive\_double

bool is\_primitive\_double() const

**Definition:** ast\_def.cpp:101

stan::gm::arg\_decl::arg\_decl

arg\_decl()

**Definition:** ast\_def.cpp:397

stan::gm::integrate\_ode::integrate\_ode

integrate\_ode()

**Definition:** ast\_def.cpp:835

stan::gm::matrix\_var\_decl::matrix\_var\_decl

matrix\_var\_decl()

**Definition:** ast\_def.cpp:1105

stan::gm::ROW\_VECTOR\_T

const int ROW\_VECTOR\_T

**Definition:** ast.hpp:68

stan::gm::print\_var\_origin

void print\_var\_origin(std::ostream &o, const var\_origin &vo)

**Definition:** ast\_def.cpp:944

stan::gm::returns\_type\_vis::return\_type\_

expr\_type return\_type\_

**Definition:** ast.hpp:689

stan::gm::function\_signatures::reset\_sigs

static void reset\_sigs()

**Definition:** ast\_def.cpp:138

stan::gm::function\_argument\_origin

const int function\_argument\_origin

**Definition:** ast.hpp:419

stan::gm::is\_user\_defined

bool is\_user\_defined(const std::string &name, const std::vector< expression > &args)

**Definition:** ast\_def.cpp:1423

stan::gm::expression::operator-=

expression & operator-=(const expression &rhs)

**Definition:** ast\_def.cpp:1391

stan::gm::no\_op\_statement

**Definition:** ast.hpp:763

stan::gm::is\_nil\_op

**Definition:** ast.hpp:270

stan::math::max

double max(const double a, const double b)

**Definition:** max.hpp:7

stan::gm::expression\_type\_vis

**Definition:** ast.hpp:199

stan::gm::variable\_dims::variable\_dims

variable\_dims()

**Definition:** ast\_def.cpp:773

stan::return\_type

Metaprogram to calculate the base scalar return type resulting from promoting all the scalar types of...

**Definition:** traits.hpp:398

stan::gm::function\_signatures::get\_signature\_matches

int get\_signature\_matches(const std::string &name, const std::vector< expr\_type > &args, function\_signature\_t &signature)

**Definition:** ast\_def.cpp:307

stan::gm::integrate\_ode

**Definition:** ast.hpp:332

stan::gm::expression::operator\*=

expression & operator\*=(const expression &rhs)

**Definition:** ast\_def.cpp:1396

stan::gm::total\_dims

size\_t total\_dims(const std::vector< std::vector< expression > > &dimss)

**Definition:** ast\_def.cpp:865

stan::gm::void\_function\_argument\_origin\_rng

const int void\_function\_argument\_origin\_rng

**Definition:** ast.hpp:424

stan::gm::array\_literal::array\_literal

array\_literal()

**Definition:** ast\_def.cpp:813

stan::gm::cov\_matrix\_var\_decl

**Definition:** ast.hpp:554

stan::gm::expression::expression

expression()

**Definition:** ast\_def.cpp:564

stan::gm::return\_statement::return\_value\_

expression return\_value\_

**Definition:** ast.hpp:758

stan::gm::function\_argument\_origin\_rng

const int function\_argument\_origin\_rng

**Definition:** ast.hpp:421

stan::gm::has\_rng\_suffix

bool has\_rng\_suffix(const std::string &s)

**Definition:** ast\_def.cpp:1406

stan::gm::fun::args\_

std::vector< expression > args\_

**Definition:** ast.hpp:352

stan::gm::function\_signatures

**Definition:** ast.hpp:106

stan::gm::function\_signatures::add\_binary

void add\_binary(const ::std::string &name)

**Definition:** ast\_def.cpp:279

stan::gm::for\_statement

**Definition:** ast.hpp:718

stan::gm::contains\_nonparam\_var

**Definition:** ast.hpp:869

stan::gm::ordered\_var\_decl::ordered\_var\_decl

ordered\_var\_decl()

**Definition:** ast\_def.cpp:1062

stan::gm::index\_op::index\_op

index\_op()

**Definition:** ast\_def.cpp:900

stan::gm::increment\_log\_prob\_statement

**Definition:** ast.hpp:712

stan::gm::printable

**Definition:** ast.hpp:256

stan::gm::print\_statement

**Definition:** ast.hpp:745

stan::gm::contains\_var::operator()

bool operator()(const nil &e) const

**Definition:** ast\_def.cpp:600

stan::gm::sample::is\_ill\_formed

bool is\_ill\_formed() const

**Definition:** ast\_def.cpp:1368

stan::gm::sample::expr\_

expression expr\_

**Definition:** ast.hpp:819

stan::gm::simplex\_var\_decl::simplex\_var\_decl

simplex\_var\_decl()

**Definition:** ast\_def.cpp:1051

stan::gm::variable\_map::get\_num\_dims

size\_t get\_num\_dims(const std::string &name) const

**Definition:** ast\_def.cpp:999

stan::gm::sample::sample

sample()

**Definition:** ast\_def.cpp:1361

stan::gm::array\_literal::args\_

std::vector< expression > args\_

**Definition:** ast.hpp:316

stan::gm::double\_literal

**Definition:** ast.hpp:307

stan::gm::double\_literal::double\_literal

double\_literal()

**Definition:** ast\_def.cpp:799

stan::gm::expression::expr\_

expression\_t expr\_

**Definition:** ast.hpp:252

stan::gm::while\_statement::body\_

statement body\_

**Definition:** ast.hpp:739

stan::gm::returns\_type\_vis::error\_msgs\_

std::ostream & error\_msgs\_

**Definition:** ast.hpp:690

stan::gm::expr\_type::num\_dims\_

size\_t num\_dims\_

**Definition:** ast.hpp:76

stan::gm::expr\_type::type

base\_expr\_type type() const

**Definition:** ast\_def.cpp:111

stan::gm::has\_non\_param\_var

bool has\_non\_param\_var(const expression &e, const variable\_map &var\_map)

**Definition:** ast\_def.cpp:748

stan::gm::unary\_op::subject

expression subject

**Definition:** ast.hpp:395

stan::gm::program::program

program()

**Definition:** ast\_def.cpp:1341

stan::gm::derived\_origin

const int derived\_origin

**Definition:** ast.hpp:417

stan::gm::operator<<

std::ostream & operator<<(std::ostream &o, const expr\_type &et)

**Definition:** ast\_def.cpp:118

stan::gm::base\_var\_decl

**Definition:** ast.hpp:428

stan::gm::conditional\_statement::bodies\_

std::vector< statement > bodies\_

**Definition:** ast.hpp:731

stan::gm::cholesky\_factor\_var\_decl

**Definition:** ast.hpp:536

stan::gm::int\_literal::type\_

expr\_type type\_

**Definition:** ast.hpp:299

stan::gm::positive\_ordered\_var\_decl

**Definition:** ast.hpp:496

stan::gm::contains\_var

**Definition:** ast.hpp:850

stan::gm::is\_linear\_function

bool is\_linear\_function(const std::string &name)

**Definition:** ast\_def.cpp:644

stan::gm::integrate\_ode::theta\_

expression theta\_

**Definition:** ast.hpp:337

stan::gm::function\_signatures::num\_promotions

int num\_promotions(const std::vector< expr\_type > &call\_args, const std::vector< expr\_type > &sig\_args)

**Definition:** ast\_def.cpp:288

stan::gm::row\_vector\_var\_decl::row\_vector\_var\_decl

row\_vector\_var\_decl()

**Definition:** ast\_def.cpp:1095

stan::gm::base\_var\_decl::base\_var\_decl

base\_var\_decl()

**Definition:** ast\_def.cpp:976

stan::gm::variable\_map

**Definition:** ast.hpp:439

stan::gm::VECTOR\_T

const int VECTOR\_T

**Definition:** ast.hpp:67

stan::gm::double\_literal::val\_

double val\_

**Definition:** ast.hpp:308

stan::gm::MATRIX\_T

const int MATRIX\_T

**Definition:** ast.hpp:69

stan::gm::promote\_primitive

expr\_type promote\_primitive(const expr\_type &et)

**Definition:** ast\_def.cpp:125

stan::gm::function\_decl\_def::function\_decl\_def

function\_decl\_def()

**Definition:** ast\_def.cpp:410

stan::gm::index\_op::type\_

expr\_type type\_

**Definition:** ast.hpp:373

stan::math::dims

void dims(const T &x, std::vector< int > &result)

**Definition:** dims.hpp:13

stan::gm::returns\_type\_vis::returns\_type\_vis

returns\_type\_vis(const expr\_type &return\_type, std::ostream &error\_msgs)

**Definition:** ast\_def.cpp:428

stan::gm::cholesky\_corr\_var\_decl::cholesky\_corr\_var\_decl

cholesky\_corr\_var\_decl()

**Definition:** ast\_def.cpp:1130

stan::gm::range::low\_

expression low\_

**Definition:** ast.hpp:402

stan::gm::expr\_type

**Definition:** ast.hpp:74

stan::gm::var\_decl::name

std::string name() const

**Definition:** ast\_def.cpp:1224

stan::gm::expression::expression\_t

boost::variant< boost::recursive\_wrapper< nil >, boost::recursive\_wrapper< int\_literal >, boost::recursive\_wrapper< double\_literal >, boost::recursive\_wrapper< array\_literal >, boost::recursive\_wrapper< variable >, boost::recursive\_wrapper< integrate\_ode >, boost::recursive\_wrapper< fun >, boost::recursive\_wrapper< index\_op >, boost::recursive\_wrapper< binary\_op >, boost::recursive\_wrapper< unary\_op > > expression\_t

**Definition:** ast.hpp:227

stan::gm::vector\_var\_decl::vector\_var\_decl

vector\_var\_decl()

**Definition:** ast\_def.cpp:1084

stan::gm::int\_literal::int\_literal

int\_literal()

**Definition:** ast\_def.cpp:781

stan::gm::contains\_nonparam\_var::contains\_nonparam\_var

contains\_nonparam\_var(const variable\_map &var\_map)

**Definition:** ast\_def.cpp:681

stan::gm::void\_function\_argument\_origin\_lp

const int void\_function\_argument\_origin\_lp

**Definition:** ast.hpp:423

stan::gm::expr\_type::num\_dims

size\_t num\_dims() const

**Definition:** ast\_def.cpp:114

stan::gm::for\_statement::statement\_

statement statement\_

**Definition:** ast.hpp:721

stan::gm::matrix\_var\_decl

**Definition:** ast.hpp:524

stan::gm::has\_var

bool has\_var(const expression &e, const variable\_map &var\_map)

**Definition:** ast\_def.cpp:675

stan::gm::binary\_op::left

expression left

**Definition:** ast.hpp:384

stan::gm::double\_var\_decl::double\_var\_decl

double\_var\_decl()

**Definition:** ast\_def.cpp:1029

stan::gm::expression

**Definition:** ast.hpp:216

stan::gm::index\_op::expr\_

expression expr\_

**Definition:** ast.hpp:371

stan::gm::binary\_op::op

std::string op

**Definition:** ast.hpp:383

stan::gm::function\_decl\_defs

**Definition:** ast.hpp:788

stan::gm::variable::type\_

expr\_type type\_

**Definition:** ast.hpp:325

stan::gm::base\_var\_decl::name\_

std::string name\_

**Definition:** ast.hpp:429

stan::gm::void\_function\_argument\_origin

const int void\_function\_argument\_origin

**Definition:** ast.hpp:422

stan::gm::simplex\_var\_decl

**Definition:** ast.hpp:480

stan::gm::expr\_type::expr\_type

expr\_type()

**Definition:** ast\_def.cpp:53

stan::gm::assignment::assignment

assignment()

**Definition:** ast\_def.cpp:1378

stan::gm::sample

**Definition:** ast.hpp:818

stan::gm::function\_signatures::add\_quaternary

void add\_quaternary(const ::std::string &name)

**Definition:** ast\_def.cpp:285

stan::gm::cholesky\_corr\_var\_decl

**Definition:** ast.hpp:546

stan::gm::reject\_statement

**Definition:** ast.hpp:751

stan::gm::model\_name\_origin

const int model\_name\_origin

**Definition:** ast.hpp:412

stan::gm::has\_lp\_suffix

bool has\_lp\_suffix(const std::string &s)

**Definition:** ast\_def.cpp:1415

stan::gm::name\_vis

**Definition:** ast.hpp:571

stan::gm::statement::statement\_t

boost::variant< boost::recursive\_wrapper< nil >, boost::recursive\_wrapper< assignment >, boost::recursive\_wrapper< sample >, boost::recursive\_wrapper< increment\_log\_prob\_statement >, boost::recursive\_wrapper< expression >, boost::recursive\_wrapper< statements >, boost::recursive\_wrapper< for\_statement >, boost::recursive\_wrapper< conditional\_statement >, boost::recursive\_wrapper< while\_statement >, boost::recursive\_wrapper< print\_statement >, boost::recursive\_wrapper< reject\_statement >, boost::recursive\_wrapper< return\_statement >, boost::recursive\_wrapper< no\_op\_statement > > statement\_t

**Definition:** ast.hpp:648

stan::gm::array\_literal::operator=

array\_literal & operator=(const array\_literal &al)

**Definition:** ast\_def.cpp:821

stan::gm::function\_signatures::key\_set

std::set< std::string > key\_set() const

**Definition:** ast\_def.cpp:380

stan::gm::reject\_statement::reject\_statement

reject\_statement()

**Definition:** ast\_def.cpp:1335

stan::gm::positive\_ordered\_var\_decl::positive\_ordered\_var\_decl

positive\_ordered\_var\_decl()

**Definition:** ast\_def.cpp:1073

stan::gm::corr\_matrix\_var\_decl::corr\_matrix\_var\_decl

corr\_matrix\_var\_decl()

**Definition:** ast\_def.cpp:1149

stan::gm::int\_var\_decl

**Definition:** ast.hpp:455

stan::gm::contains\_var::contains\_var

contains\_var(const variable\_map &var\_map)

**Definition:** ast\_def.cpp:597

stan::gm::double\_literal::type\_

expr\_type type\_

**Definition:** ast.hpp:309

stan::gm::variable\_map::get\_base\_type

base\_expr\_type get\_base\_type(const std::string &name) const

**Definition:** ast\_def.cpp:996

ast.hpp

stan::gm::contains\_nonparam\_var::operator()

bool operator()(const nil &e) const

**Definition:** ast\_def.cpp:684

stan::gm::transformed\_data\_origin

const int transformed\_data\_origin

**Definition:** ast.hpp:414

stan::gm::base\_expr\_type

int base\_expr\_type

**Definition:** ast.hpp:60

stan::gm::is\_nil

bool is\_nil(const expression &e)

**Definition:** ast\_def.cpp:768

stan::gm::statement::statement\_

statement\_t statement\_

**Definition:** ast.hpp:650

stan::math::e

double e()

Return the base of the natural logarithm.

**Definition:** constants.hpp:86

stan::gm::var\_decl::decl\_

var\_decl\_t decl\_

**Definition:** ast.hpp:609

stan::gm::fun

**Definition:** ast.hpp:350

stan::gm::printable::printable

printable()

**Definition:** ast\_def.cpp:589

stan::gm::array\_literal::type\_

expr\_type type\_

**Definition:** ast.hpp:317

stan::gm::expr\_type::operator!=

bool operator!=(const expr\_type &et) const

**Definition:** ast\_def.cpp:70

stan::gm::sample::truncation\_

range truncation\_

**Definition:** ast.hpp:821

stan::math::size

int size(const std::vector< T > &x)

**Definition:** size.hpp:11

stan::gm::expr\_type::is\_ill\_formed

bool is\_ill\_formed() const

**Definition:** ast\_def.cpp:105

stan::gm::unit\_vector\_var\_decl

**Definition:** ast.hpp:472

stan::gm::fun::fun

fun()

**Definition:** ast\_def.cpp:853

stan::gm::returns\_type\_vis

**Definition:** ast.hpp:688

stan::gm::variable\_map::remove

void remove(const std::string &name)

**Definition:** ast\_def.cpp:1012

stan::gm::name\_vis::name\_vis

name\_vis()

**Definition:** ast\_def.cpp:1160

stan::gm::binary\_op

**Definition:** ast.hpp:382

stan::gm::arg\_decl::base\_variable\_declaration

base\_var\_decl base\_variable\_declaration()

**Definition:** ast\_def.cpp:403

stan::gm::range::has\_high

bool has\_high() const

**Definition:** ast\_def.cpp:940

stan::gm::is\_assignable

bool is\_assignable(const expr\_type &l\_type, const expr\_type &r\_type, const std::string &failure\_message, std::ostream &error\_msgs)

**Definition:** ast\_def.cpp:1453

stan::gm::infer\_type\_indexing

expr\_type infer\_type\_indexing(const base\_expr\_type &expr\_base\_type, size\_t num\_expr\_dims, size\_t num\_index\_dims)

**Definition:** ast\_def.cpp:873

stan::gm::is\_nil\_op::operator()

bool operator()(const nil &x) const

**Definition:** ast\_def.cpp:754

stan::gm::function\_argument\_origin\_lp

const int function\_argument\_origin\_lp

**Definition:** ast.hpp:420

stan::gm::range::range

range()

**Definition:** ast\_def.cpp:931

stan::gm::INT\_T

const int INT\_T

**Definition:** ast.hpp:65

stan::gm::expr\_type::operator>

bool operator>(const expr\_type &et) const

**Definition:** ast\_def.cpp:83

stan::gm::unary\_op::unary\_op

unary\_op(char op, expression const &subject)

**Definition:** ast\_def.cpp:923

stan::gm::index\_op::infer\_type

void infer\_type()

**Definition:** ast\_def.cpp:907

stan::gm::return\_statement

**Definition:** ast.hpp:757

stan::gm::expression::operator+=

expression & operator+=(const expression &rhs)

**Definition:** ast\_def.cpp:1386

stan::gm::variable

**Definition:** ast.hpp:323

stan::gm::binary\_op::binary\_op

binary\_op()

**Definition:** ast\_def.cpp:911

stan::gm::expression\_type\_vis::operator()

expr\_type operator()(const nil &e) const

**Definition:** ast\_def.cpp:533

stan::gm::transformed\_parameter\_origin

const int transformed\_parameter\_origin

**Definition:** ast.hpp:416

stan::gm::expr\_type::operator<

bool operator<(const expr\_type &et) const

**Definition:** ast\_def.cpp:73

stan::gm::cholesky\_factor\_var\_decl::cholesky\_factor\_var\_decl

cholesky\_factor\_var\_decl()

**Definition:** ast\_def.cpp:1118

stan::gm::is\_no\_op\_statement\_vis

**Definition:** ast.hpp:671

stan::gm::statements::statements

statements()

**Definition:** ast\_def.cpp:526

stan::gm::expr\_type::operator<=

bool operator<=(const expr\_type &et) const

**Definition:** ast\_def.cpp:78

stan::gm::double\_literal::operator=

double\_literal & operator=(const double\_literal &dl)

**Definition:** ast\_def.cpp:806

stan::gm::variable\_map::range\_t

std::pair< base\_var\_decl, var\_origin > range\_t

**Definition:** ast.hpp:440

stan::gm::vector\_var\_decl

**Definition:** ast.hpp:504

stan::gm::name\_vis::operator()

std::string operator()(const nil &x) const

**Definition:** ast\_def.cpp:1161

stan::gm::function\_signatures::get\_result\_type

expr\_type get\_result\_type(const std::string &name, const std::vector< expr\_type > &args, std::ostream &error\_msgs)

**Definition:** ast\_def.cpp:329

stan::gm::while\_statement

**Definition:** ast.hpp:737

stan::gm::unit\_vector\_var\_decl::unit\_vector\_var\_decl

unit\_vector\_var\_decl()

**Definition:** ast\_def.cpp:1040

stan::gm::variable::name\_

std::string name\_

**Definition:** ast.hpp:324

stan::math::dist

double dist(const std::vector< double > &x, const std::vector< double > &y)

**Definition:** dist.hpp:11

stan::gm::int\_literal::val\_

int val\_

**Definition:** ast.hpp:298

stan::gm::variable\_map::exists

bool exists(const std::string &name) const

**Definition:** ast\_def.cpp:988

stan::gm::write\_base\_expr\_type

std::ostream & write\_base\_expr\_type(std::ostream &o, base\_expr\_type type)

**Definition:** ast\_def.cpp:23

stan::gm::expression::expression\_type

expr\_type expression\_type() const

**Definition:** ast\_def.cpp:570

stan::gm::var\_decl::var\_decl\_t

boost::variant< boost::recursive\_wrapper< nil >, boost::recursive\_wrapper< int\_var\_decl >, boost::recursive\_wrapper< double\_var\_decl >, boost::recursive\_wrapper< vector\_var\_decl >, boost::recursive\_wrapper< row\_vector\_var\_decl >, boost::recursive\_wrapper< matrix\_var\_decl >, boost::recursive\_wrapper< simplex\_var\_decl >, boost::recursive\_wrapper< unit\_vector\_var\_decl >, boost::recursive\_wrapper< ordered\_var\_decl >, boost::recursive\_wrapper< positive\_ordered\_var\_decl >, boost::recursive\_wrapper< cholesky\_factor\_var\_decl >, boost::recursive\_wrapper< cholesky\_corr\_var\_decl >, boost::recursive\_wrapper< cov\_matrix\_var\_decl >, boost::recursive\_wrapper< corr\_matrix\_var\_decl > > var\_decl\_t

**Definition:** ast.hpp:607

stan::gm::variable\_map::get

base\_var\_decl get(const std::string &name) const

**Definition:** ast\_def.cpp:991

stan::gm::conditional\_statement::conditional\_statement

conditional\_statement()

**Definition:** ast\_def.cpp:1315

stan::gm::returns\_type

bool returns\_type(const expr\_type &return\_type, const statement &statement, std::ostream &error\_msgs)

**Definition:** ast\_def.cpp:515

stan::gm::variable::set\_type

void set\_type(const base\_expr\_type &base\_type, size\_t num\_dims)

**Definition:** ast\_def.cpp:829

stan::gm::contains\_nonparam\_var::var\_map\_

const variable\_map & var\_map\_

**Definition:** ast.hpp:870

stan::gm::int\_literal::operator=

int\_literal & operator=(const int\_literal &il)

**Definition:** ast\_def.cpp:792

stan::gm::arg\_decl::arg\_type\_

expr\_type arg\_type\_

**Definition:** ast.hpp:768

stan::gm::var\_origin

int var\_origin

**Definition:** ast.hpp:411

stan::gm::returns\_type\_vis::operator()

bool operator()(const nil &st) const

**Definition:** ast\_def.cpp:433

stan::gm::function\_signatures::add\_ternary

void add\_ternary(const ::std::string &name)

**Definition:** ast\_def.cpp:282

stan::gm::statements::statements\_

std::vector< statement > statements\_

**Definition:** ast.hpp:187

stan::gm::expr\_type::is\_primitive

bool is\_primitive() const

**Definition:** ast\_def.cpp:93

stan::gm::int\_var\_decl::int\_var\_decl

int\_var\_decl()

**Definition:** ast\_def.cpp:1016

stan::gm::statement::statement

statement()

**Definition:** ast\_def.cpp:1228

stan::gm::variable\_map::get\_origin

var\_origin get\_origin(const std::string &name) const

**Definition:** ast\_def.cpp:1002

stan::gm::contains\_var::var\_map\_

const variable\_map & var\_map\_

**Definition:** ast.hpp:851

stan::gm::return\_statement::return\_statement

return\_statement()

**Definition:** ast\_def.cpp:1324

stan::gm::index\_op

**Definition:** ast.hpp:370

stan::gm::ILL\_FORMED\_T

const int ILL\_FORMED\_T

**Definition:** ast.hpp:70

stan::gm::expr\_type::is\_void

bool is\_void() const

**Definition:** ast\_def.cpp:108

M\_

int M\_

**Definition:** mdivide\_left.hpp:19

stan::gm::VOID\_T

const int VOID\_T

**Definition:** ast.hpp:64

stan::gm::for\_statement::for\_statement

for\_statement()

**Definition:** ast\_def.cpp:1297

stan::gm::while\_statement::while\_statement

while\_statement()

**Definition:** ast\_def.cpp:1307

stan::gm::is\_no\_op\_statement\_vis::operator()

bool operator()(const nil &st) const

**Definition:** ast\_def.cpp:1246

stan::gm::function\_signature\_t

std::pair< expr\_type, std::vector< expr\_type > > function\_signature\_t

**Definition:** ast.hpp:104


---

[ Stan Home Page ]

*© 2011–2014,
Stan Development Team.*
